# Fatal pulmonary arterial thrombosis in a COVID-19 patient, with asymptomatic history, occurred after swab negativization

**DOI:** 10.1186/s12959-020-00255-6

**Published:** 2021-01-06

**Authors:** Franca Del Nonno, Daniele Colombo, Roberta Nardacci, Laura Falasca

**Affiliations:** 1 Pathology Unit, National Institute for Infectious Diseases “L. Spallanzani”, IRCCS, Rome, Italy; 2 Laboratory of Electron Microscopy, National Institute for Infectious Diseases “L. Spallanzani”, IRCCS, Rome, Italy

**Keywords:** COVID-19, SARS-CoV-2, Asymptomatic, Thrombosis, Lung, Heart

## Abstract

**Background:**

A considerable number of SARS-CoV-2 infected individuals could be asymptomatic and don’t need medical treatment. The clinical spectrum of SARS-CoV-2 infection ranges from asymptomatic cases, medium-intensity forms with mild to moderate symptoms, to severe ones with bilateral pneumonia and respiratory distress. In cases with severe presentation of SARS-CoV-2 infection, the induction of hypercoagulability is one of the pathophysiological mechanism that can contribute to death.

**Case presentation:**

Here, we reported autoptic evidences of thrombotic pulmonary arterial fatal lesions in an asymptomatic COVID-19 patient, after swab negativization. Whole body complete post-mortem examination was performed, showing the presence of a large thrombus occluding the main pulmonary artery that was the cause of death. Histopathological analysis showed heterogeneous pattern of pathological changes in the lung tissue with numerous vascular thrombi, inflammatory cardiomyopathy and other histopathological modifications in kidneys, spleen and liver.

**Conclusions:**

This study provides evidences that also asymptomatic patients may be at risk to develop thrombotic complications. An appropriate diagnostic screening for thrombotic complications and the early treatment recommendations of antithrombotic drugs could represent an important topic even in asymptomatic individuals.

## Introduction

Asymptomatic COVID-19 cases are those having positive results for SARS-CoV-2 RNA but show no signs of illness. SARS-CoV-2 infection is usually associated with a large-spectrum clinical presentation, which classically involves the respiratory tract. Acute respiratory distress syndrome and multiple organ failure are features of severe cases of COVID-19 [1]. Hypercoagulable condition, accompanied by thrombosis and disseminated intravascular coagulation, may determine progression to multiple-organ failure and death [2]. The association of COVID-19 with clinically significant coagulopathies and multiple infarcts has been described [3], thus an appropriate evaluation and interventions to prevent and treat thromboembolic complications in patients showing coagulopathy, is matter of interest [4].

Here we presented the occurrence of fatal pulmonary arterial thrombosis in an asymptomatic COVID-19 patient.

## Case presentation

A 61-year-old woman was referred to an emergency department in Rome (Italy) due to sudden loss of consciousness and cardiac arrest. She could not be resuscitated and was declared dead soon after admission. The patient had contact history with confirmed COVID-19 patients, and 32 days before decease was tested positive for SARS-CoV-2 real-time polymerase chain reaction (RT-PCR), from a nasopharyngeal swab. Patient tested negative 5 days before death; the test was repeated at a distance of 48 h, confirming negative result. She had no medical co-morbidities or any cause of immunosuppression, taking no medications, and presented as healthy individual before SARS-CoV-2 infection. No relevant symptoms have been shown neither at the time of RT-PCR test, nor until exitus.

To determine the cause of death a whole body post-mortem examination was performed at the National Institute for Infectious Diseases Lazzaro Spallanzani-IRCCS Hospital (Rome, Italy).

**Fig. 1:**
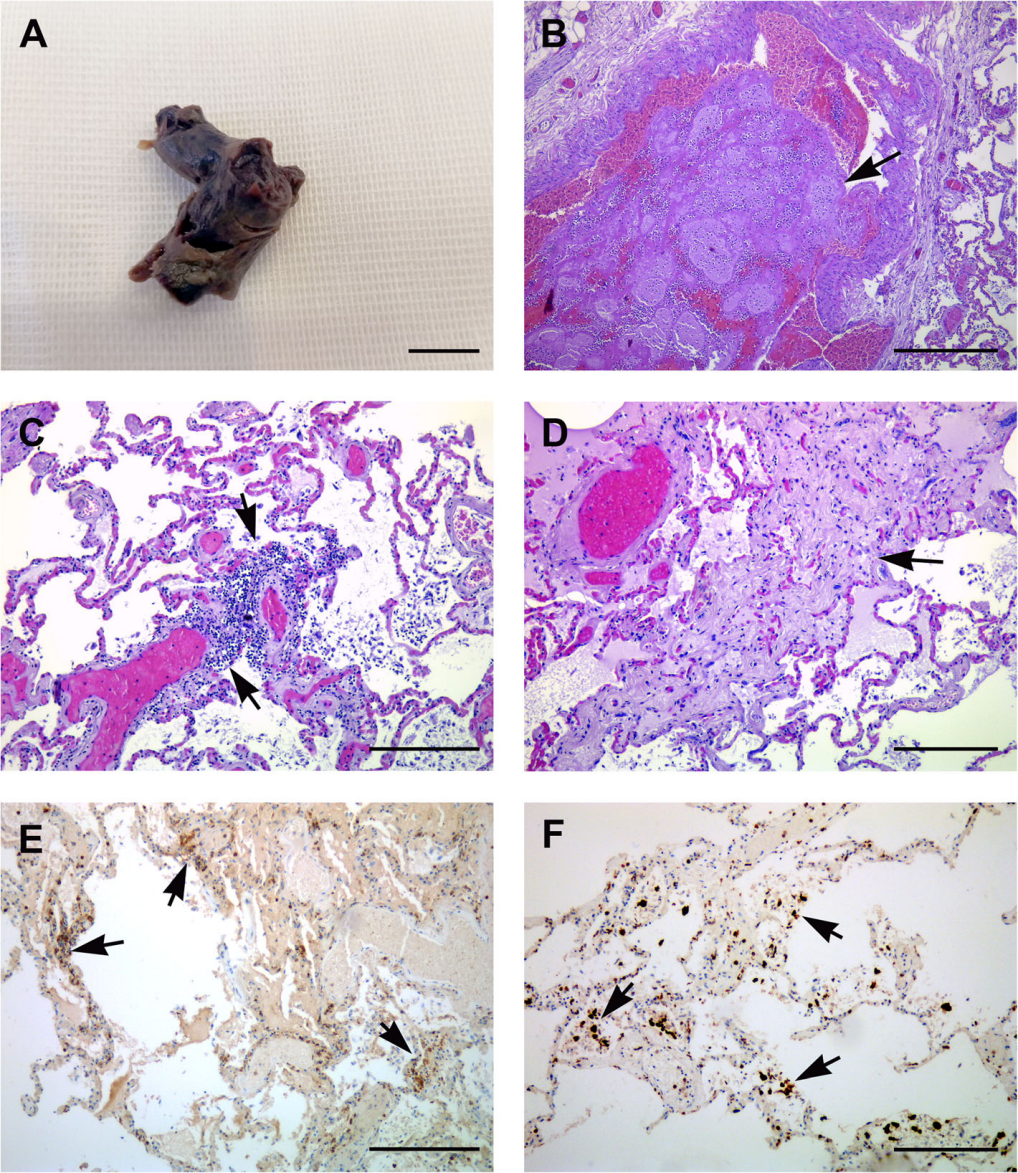
Pathological findings. **a** Gross pathological specimen of the thrombus occluding pulmonary artery bilaterally. The specimen consists of an irregular fragment of red-tan hemorrhagic tissue measuring about 1.3 cm in diameter. **b** Light microscopy of lung tissue section shows an intravascular thrombus of large vessel (arrow). **c** Lung parenchyma showing inflammatory cells in the pulmonary interstitium (arrows) and in alveolar space. **d** Lung diffuse interstitial fibrosis is visible (arrow). Numerous CD4+ T lymphocytes (**e**) and CD68+ macrophages (**f**) are diffusely present into alveolar septa and around vessels (arrows). Scale bars: **a** = 1 cm; **b** = 100 μm; **c**-**f** = 50 μm

**Fig. 2:**
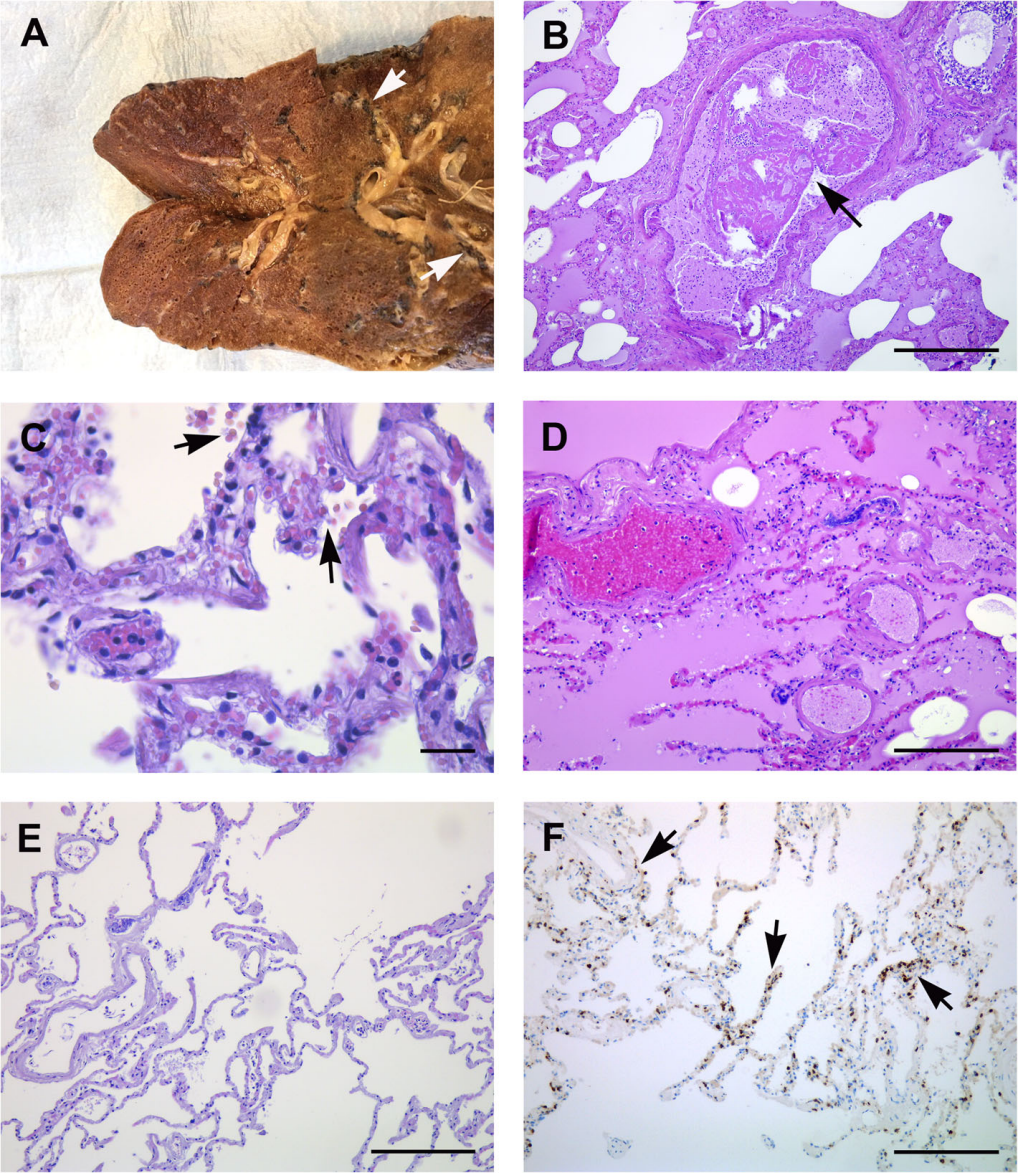
Histological characterization of lung tissue. **a** Gross examination of the lungs shows massive thrombosis of medium-sized arteries (arrows). **b** Lung tissue section display a large vessel partially obstructed by a thrombus (arrow). **c** Lung parenchyma showing extravasated eritrocytes in interalveolar septa (arrows). **d** Edema and inflammatory infiltrate are visible in multiple foci in the lung tissue. **e** Lung areas not affected by histopathological changes. **f** Numerous CD8+ T lymphocytes are present into alveolar septa and around vessels (arrows). Scale bars: **b** = 100 μm; **c** = 7 μm; **d-f** = 50 μm

Macroscopic inspection of the lungs revealed pulmonary edema, massive bilateral congestion and regions of dark-colored hemorrhage. The pleura was inconspicuous, except for fibrous adhesions, and pleural effusion was absent. The most striking feature was the presence of a large thrombus occluding the main pulmonary artery at bifurcation (Fig. 1a). The presence of thrombi was detected also in medium-sized arteries (Fig. 2a). Lungs were bilaterally extensively sampled for a complete histological evaluation. Microscopic analysis of tissue showed heterogeneous pattern of pathological changes and different stages of diffuse alveolar damage, with edema and hemorrhagic areas (Fig. 2d). Numerous vascular thrombi were detected (Figs. 1b; 2b). Diffuse interstitial fibrosis with fibroblast proliferation was present (Fig. 1d), together with inflammatory infiltrate in pulmonary interstitium (Figs. 1c; 2d). The alveolar capillaries were thickened, and displayed extravasation of erythrocytes into alveolar spaces (Fig. 2c). Infiltrating T lymphocytes, both CD4+ (Fig. 1e) and CD8+ (Fig. 2f), were found into alveolar septa and clustered around capillary vessels, as demonstrated by immunohistochemistry. In addition numerous macrophages (CD68+) were present (Fig. 1f). Of note, positivity for CD20 was not detected, indicating the absence of infiltrating B-lymphocytes. Some areas of the lungs appeared not affected and presented no signs of alveolar damage (Fig. 2e).

**Fig. 3:**
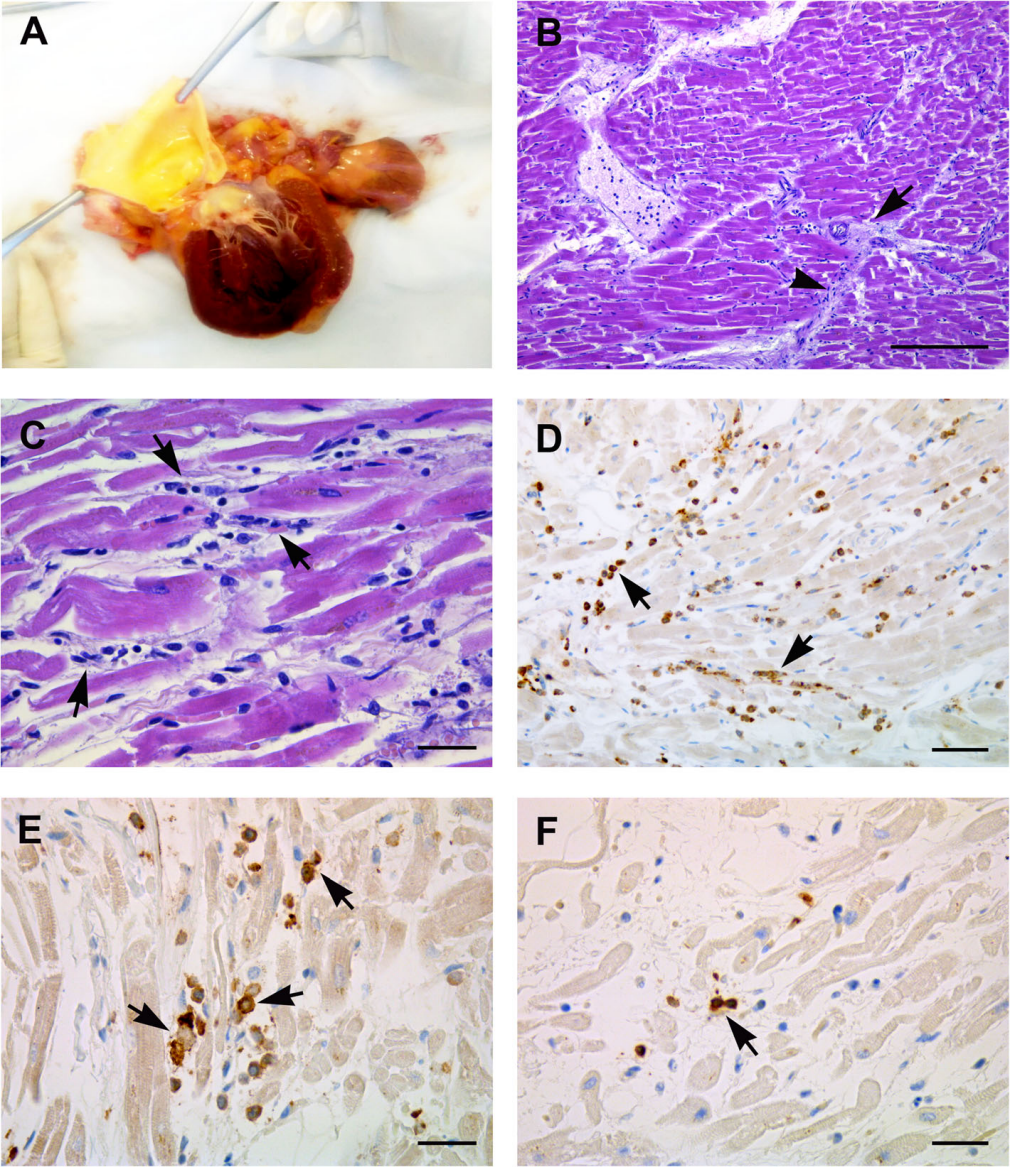
Pathological findings of heart. **a** Macroscopic inspection shows decreased volume and consistency of the heart. **b** Histological analysis showing interstitial inflammatory infiltrate and variable degrees of interstitial (arrowhead) and vascular (arrow) fibrosis. **c** Heart tissue shows myocarditis characterized by mononuclear, predominantly lymphocytic infiltrate (arrows). **d** The immune-characterization of inflammatory cells shows that numerous macrophages (CD68+) infiltrates the myocardium (arrows). CD4+ T lymphocytes are mostly represented (**e**) while CD8+ T lymphocytes are rare (**f**) (arrows). Scale bars: **b** = 100 μm; **d** = 14 μm; **c**,**e**,**f** = 7 μm

Gross examination of heart revealed decrease in volume and consistency (weight 250 g). Left and right atrium and ventricles appeared dilated. The myocardium was flabby, congested and hemorrhagic (Fig. 3a). At the histological level, myocytes hypertrophy and variable degrees of interstitial and vascular fibrosis were found (Fig. 3b). Active myocarditis was characterized predominantly by lymphocytic mononuclear infiltrate dissociating myocyte fibers (Fig. 3c). The infiltrating cells were mainly represented by CD68+ macrophages (Fig. 3d). CD4+ T lymphocytes were numerous (Fig. 3e), while CD8+ T lymphocytes were rare (Fig. 3f); B-lymphocytes were not detected.

Other organs analyzed showed histopathological modifications, similarly to findings reported in autopsy of severe COVID-19 patients [1]: kidneys displayed interstitial and perivascular fibrosis; the spleen showed white pulp atrophy and congestion of red pulp; macrovesicular steatosis was observed in the liver (data not shown).

In the final report, the cause of death was listed as pulmonary arterial thrombosis.

## Discussion and conclusions

A considerable number of SARS-Cov-2 infected individuals could be asymptomatic, i.e. having viral nucleic acid or antibody testing positive, but without displaying any symptom [5]. A multi-center retrospective study, based on 100 individuals with asymptomatic infection, reported that 60% of cases demonstrated findings of pneumonia by chest CT imaging, including well-recognized features of coronavirus disease, such as ground-glass opacities [6].

To our knowledge, this is the first report describing autoptic evidences of COVID-19 thrombotic fatal lesions in a case of asymptomatic SARS-CoV-2 infection.

The induction of hypercoagulability has recognized as one of the pathophysiological mechanism in patients with severe presentation of the SARS-CoV-2 infection [7]. Coagulation disorders determine an increased risk of thrombotic complications and predisposes patients to a greater chance of mortality [8].

The risk for deep vein thrombosis and pulmonary embolism in COVID-19 patients is in part attributable to hypoxia and immobilization in intensive care unit [9, 10]. In addition, it has been suggested that SARS-CoV-2 infection could affect the coagulation cascade and fibrinolysis either directly, by producing vascular dysfunction through viral effect on endothelial cells, or indirectly, by exacerbated induction of inflammatory cytokines (e.g. tumor necrosis factor-α (TNF-α) and interleukin-6 (IL-6) [11]. An exaggerated inflammatory response may induce a condition in which coagulation contributes to pathological arterial thrombotic events [12].

Results obtained by detailed autopsy studies of COVID-19 patients, played a key role in understanding organ damage and revealed vascular involvement, showing large vessel and microvascular thrombosis, pulmonary hemorrhage and disseminated intravascular coagulation in different organs [1, 13, 14].

Pathological findings of this study showed that sudden death was attributable to pulmonary thrombosis, associated with interstitial fibrosis and hemorrhagic destruction of the lung parenchyma. Lung histopathological changes were similar to those we previously described in post-mortem lung tissues from 22 COVID-19 patients [1], thus indicating that asymptomatic patients can develop hidden lung pathology. Accordingly, a recently published case series displays that computerized tomography (CT) can reveal lung pathology in some asymptomatic and pauci-symptomatic patients [15].

The inflammation of cardiac interstitium could also have contributed to the unexpected death. Cardiovascular complications, such as heart failure, myocarditis, pericarditis, vasculitis, and cardiac arrhythmias has been reported in COVID-19 in patients without preexisting cardiovascular diseases. Even in some patients who recover, inflammatory cardiomyopathy could persist [16].

No data are available on the risk of thromboembolism in non-hospitalized COVID-19 patients. The American Society of Hematology recommended the use of prophylactic dose of low molecular weight heparin for all patients with COVID-19, who did not have a contraindication for it. However, the empiric use of a therapeutic dose of anticoagulation in COVID-19 patients is still an open question. The International Society of Thrombosis and Haemostasis (ISTH) interim guidance, recommends monitoring of coagulation parameters such as D-dimer, fibrinogen, prothrombin time, and platelet count, in assisting the use of antithrombotic drugs in hospitalized patients with COVID-19 [17, 18]. The worsening of coagulation parameters may warrant for administration pharmacologic thromboprophylaxis.

A limit of this study is the lack of knowledge of the elevation of hypercoagulability markers, due to patient’s sudden death. The merit of our study is to provide evidences that also asymptomatic COVID-19 patients may develop thrombotic complications suggesting that an appropriate diagnostic screening for thrombotic complications and to the early treatment recommendations of antithrombotic drugs could represent an important topic.

## Data Availability

Via correspondence with RN.
